# Computational identification of potential inhibitors targeting *cdk1* in colorectal cancer

**DOI:** 10.3389/fchem.2023.1264808

**Published:** 2023-11-30

**Authors:** Uchechukwu C. Ogbodo, Ojochenemi A. Enejoh, Chinelo H. Okonkwo, Pranavathiyani Gnanasekar, Pauline W. Gachanja, Shamim Osata, Halimat C. Atanda, Emmanuel A. Iwuchukwu, Ikechukwu Achilonu, Olaitan I. Awe

**Affiliations:** ^1^ Department of Applied Biochemistry, Nnamdi Azikiwe University, Awka, Nigeria; ^2^ Genomics and Bioinformatics Department, National Biotechnology Development Agency, Abuja, Nigeria; ^3^ Department of Pharmacology and Toxicology, University of Nigeria, Nsukka, Nigeria; ^4^ Department of Bioinformatics, Pondicherry University, Puducherry, India; ^5^ Department of Biochemistry and Biotechnology, Pwani University, Kilifi, Kenya; ^6^ Department of Biochemistry, University of Nairobi, Nairobi, Kenya; ^7^ Biotechnology Department, Federal University of Technology, Akure, Nigeria; ^8^ Protein Structure-Function Research Unit, School of Molecular and Cell Biology, Faculty of Sciences, University of Witwatersrand, Johannesburg, South Africa; ^9^ Department of Computer Science, University of Ibadan, Ibadan, Nigeria; ^10^ African Society for Bioinformatics and Computational Biology, Cape Town, South Africa

**Keywords:** colorectal cancer, cyclin-dependent kinase 1, inhibitors, natural compounds, molecular dynamics simulation

## Abstract

**Introduction:** Despite improved treatment options, colorectal cancer (CRC) remains a huge public health concern with a significant impact on affected individuals. Cell cycle dysregulation and overexpression of certain regulators and checkpoint activators are important recurring events in the progression of cancer. Cyclin-dependent kinase 1 (CDK1), a key regulator of the cell cycle component central to the uncontrolled proliferation of malignant cells, has been reportedly implicated in CRC. This study aimed to identify CDK1 inhibitors with potential for clinical drug research in CRC.

**Methods:** Ten thousand (10,000) naturally occurring compounds were evaluated for their inhibitory efficacies against CDK1 through molecular docking studies. The stability of the lead compounds in complex with CDK1 was evaluated using molecular dynamics simulation for one thousand (1,000) nanoseconds. The top-scoring candidates’ ADME characteristics and drug-likeness were profiled using SwissADME.

**Results:** Four hit compounds, namely, spiraeoside, robinetin, 6-hydroxyluteolin, and quercetagetin were identified from molecular docking analysis to possess the least binding scores. Molecular dynamics simulation revealed that robinetin and 6-hydroxyluteolin complexes were stable within the binding pocket of the CDK1 protein.

**Discussion:** The findings from this study provide insight into novel candidates with specific inhibitory CDK1 activities that can be further investigated through animal testing, clinical trials, and drug development research for CRC treatment.

## 1 Introduction

Colorectal cancer (CRC) is the third most common cancer next to lung and breast cancers with an estimated diagnosis of 2 million individuals and a mortality of approximately 900,000 annually (Bray et al., 2020; Holowatyj et al., 2020). It is thought to be a disease in high-income countries; however, studies have shown the incidence of CRC to increase at a projected rate of 70% by 2030 for low and middle-income countries (Ferlay et al., 2015) with a 5% lifetime average-risk incidence globally (Weitz et al., 2005). These gory statistics have significant implications for the public health systems both in the country and globally hence the need for a more dynamic scientific approach to the CRC burden.

The onset of CRC follows a pattern of abnormal growth of the epithelial cells of the colon and/or rectum leading to a tumor mass that fills the intestinal lumen, metastasizing into nearby organs of the abdominal area, lymph nodes, and other parts of the body (Adeoti et al., 2016). It usually begins as a small, adenomatous mass of cells known as polyps that form on the inside of the colon, which may become malignant or remain benign (Ikwu et al., 2020). As with many cancers, the cause of CRC is not known (Simon et al., 2017), however, risk factors such as age, lifestyle habits (alcohol and tobacco use), high-fat diet, predisposing medical conditions such as type 2 diabetes, and a family history of CRC have been associated with the disease course. Although traditional chemotherapy options have yielded remarkable results in CRC treatment with possibilities of relapses, limited molecular understanding of the oncogenetic initiation and interplay of biochemical mechanisms/factors have impaired the development of novel pharmacologically therapeutic anti-cancer drug agents.

**FIGURE 1:**
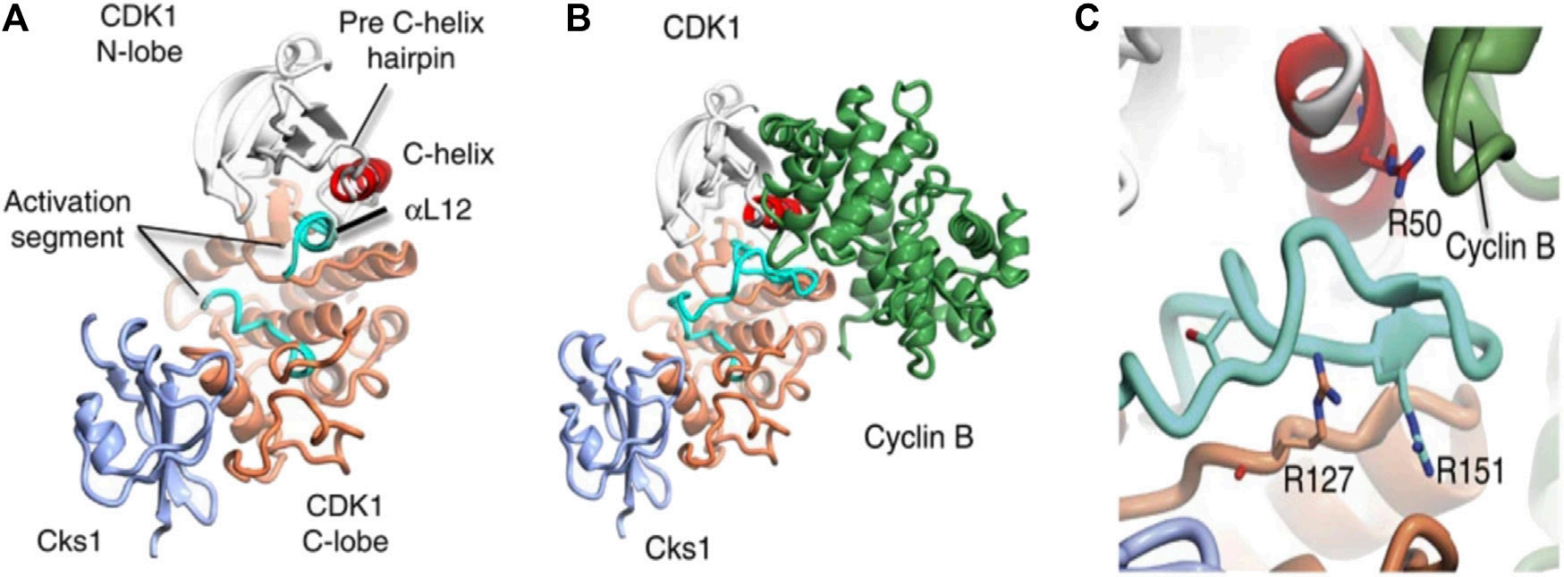
Structure of CDKI binding to CKs1 and complexation with cyclin B. The complexes shown above depict **(A)** CDK1-Cks1 **(B)** CDK1-cyciln B-Cks1 **(C)** residues of CDK1. In each image, the following colours apply; CDKI N-lobe (white), CDK1 C- lobe (coral), Cks subunit (ice-blue), C-helix (red), activation segment (cyan), cyclin subunit (lawn green) (Brown et al., 2015).

Cyclin-dependent kinase 1 (CDK1) enzyme has been implicated in the oncogenesis of CRC (Sofi et al., 2022a). It is a serine/threonine kinase known to be the catalytic subunit of the M-phase promoting factor (MPF) complex (Kalous et al., 2020) and belongs to a family of cyclic cell regulatory proteins involved in maintaining cell cycle efficiency (Sofi et al., 2022a; Sofi et al., 2022b). Though involved in many cellular events that ensure cell survival, CDK1 specifically acts by regulating the G_2_/M and G_1_/S phases oscillating in activity between the M transition stages to bring about replication, activating checkpoints, and more recently has been essential to replication fork maintenance (Adhikari et al., 2012; Liao et al., 2017; Matthews et al., 2022). Prior to initiating cell replication, CDK1 is activated by binding to cyclin B_1_ to form CDK1-cyclin B complex (Figure 1) (Brown et al., 2015). However, the over expression of CDK1 has been linked to unrestricted cell proliferation. Its over expression has been linked to unrestricted cell proliferation, tumor development, and dysregulation associated with the initiation of many cancers including colorectal cancer (Li et al., 2020; Sofi et al., 2022a; Sofi et al., 2022b). Gene expression has been associated with biomarkers in complex diseases like prostate cancer (Chikwambi et al., 2023), epilepsy (El Abed et al., 2023), malaria/COVID-19 (Nzungize et al., 2022) and SARS-CoV-2 infection (Nyamari et al., 2023). Several studies suggest that overexpression of CDK1, which is the hallmark event in many malignancies is associated with 5 = fluorouracil resistance through altered activation of Wnt/β-catechin signaling, mTOR, and p53 pathways leading to poor prognosis in CRC (Zhu et al., 2019; Zhu et al., 2020a; Zhu et al., 2020b; Sofi et al., 2022a).

Natural products have drawn a lot of attention recently as potential sources of treatments for a variety of illnesses. In recent years, bioactive compounds from plants have been proven to have opportunities in the pharmaceutical industry for therapeutic purposes (Sasidharan et al., 2011). Resveratrol, a polyphenol has been documented for its ability to reduce or inhibit cell proliferation, reduce apoptosis and suppress angiogenesis and metastasis in cancer cells (Greenlee et al., 2022; Dar et al., 2023). Also, flavopiridol, a flavonoid based substance has undergone clinical trials for its anti-cancer benefits and as a potent Cyclin-Dependent Kinase (CDK) inhibitor (Deep et al., 2018). By specifically targeting CDK1, cell cycle arrest is initiated, slowing down the growth of tumors. Compared to synthetic alternatives, natural products have fewer adverse effects.

Despite recent advances in diagnostics and treatments for CRC as well as the failure of conventional cancer treatments including chemotherapy and radiotherapy to sustain lower relapse rates, there remains a gap in treatment and increasing incidence of CRC cases. With the vast plethora of computer-aided drug design and combinatorial chemistry technologies available, a potentially wide array of therapeutic options from small molecule compounds that can target significant aspects of CDK1 activity exists. Our study investigated potential inhibitors of CDK1 *in silico* and explored their pharmacokinetic properties using a number of computational techniques.

## 2 Materials and methods

### 2.1 Ligand preparation

A library of ten thousand bioactive plant compounds was compiled and downloaded from the PubChem database (https://pubchem.ncbi.nlm.nih.gov). Pubchem is a publicly available small molecule database (Li et al., 2010; Gozalbes and Pineda-Lucena, 2011; Cheng et al., 2014) used in drug discovery and other applications. It contains information on over 250 million unique chemical molecules along with their descriptors. Before carrying out docking, the two-dimensional (2D) structure data file (SDF) format of the compounds downloaded from the pubchem repository were imported into Maestro (Schrodinger suite) for transformation in the LigPrep module of the software (Epik, 2017a). Ionization states were generated at a pH range of 7 ± 2.0 and stereoisomers were calculated at default (Sastry et al., 2013).

### 2.2 Protein preparation

The three-dimensional (3D) human crystal X-ray diffraction structure of Cyclin-dependent kinase 1 (http://www.rcsb.org/pdb/home; PDB ID: 4Y72) (Brown et al., 2015), was downloaded as a pdb file and imported into Maestro suite. For appropriate structural preparation of the virtual protein using the Protein Preparation wizard of the Schrodinger software, hydrogen, bond orders, and missing side chains were added appropriately while water molecules beyond 5 Å from het groups were deleted before optimization at pH 7.0 + 2.0. The protein structure was subsequently minimized using the optimized potentials for liquid simulations (OPLS) 2005 force field to a root-mean-square-deviation (RMSD) of 0.30 Å (Epik, 2017b).

### 2.3 Molecular docking

The compounds were subjected to molecular docking within the active site of CDK1. Using the receptor grid generation feature in the Schrodinger suite, a grid box where each ligand will be docked was generated. The binding pocket where the co-crystallized ligand is located was used to construct the defining box as well as the residue locations within 4 Å radius of the ligand. Molecular docking was performed via the standard precision (SP) and extra precision (XP) of the Glide tool in the software (Friesner et al., 2006; Tripathi et al., 2013). Following docking, the top compounds with optimal binding scores were selected for further studies.

### 2.4 Drug likeness and *in silico* ADME predictions

The drug-likeness of the lead compounds was evaluated by subjecting them to computational pharmacological and pharmacokinetic analyses on the SwissADME web server (http://www.swissadme.ch) (Daina et al., 2017; Guan et al., 2019), using the generated Canonical SMILES (simplified molecular-input entry system) transformations of the structures (Cheng et al., 2022). Lipinski’s rule of five was used to assess the compounds based on the following parameters of molecular weight, number of hydrogen bond donors and acceptors and octanol-water partition (Lipinski et al., 1997; Veber et al., 2002; Yang et al., 2019). Pro-Tox II online server (https://tox-new.charite.de/protox_II) was used to predict the toxicity of the compounds (Banerjee et al., 2018).

### 2.5 Induced fit docking

The top four ligands with the least binding scores were selected and subjected to the mixed molecular docking and dynamic protocol used by the induced-fit docking (IFD) module of the Maestro algorithm. An implicit solvent model with the OPLS 2005 force field was used to apply the standard IFD protocol to the chosen (centroid) amino acid side chains (8–10, 18–20, 31–33, 64, 80–89, 135, 145–146). The IFD procedure included a ring conformational sampling with a 2.5 kcal/mol energy barrier and a non-planar conformation penalty on amide bonds. Each ligand was given a maximum of 20 permitted postures, with the scaling for both the receptor and the ligand fixed at 0.5. The Prime Refinement technique was used to further refine residues that were within 5 Å of the docked ligand. The protein-ligand complexes with the improved structure were ranked using prime energy. For a final round of Glide docking and scoring, the receptor structures that were less than 30 kcal/mol from the minimal energy structure were submitted. In the next second docking stage, all ligands were re-docked into all revised low-energy receptor structures using Glide XP’s default settings.

### 2.6 Molecular dynamics simulation

The docked complexes were further subjected to molecular dynamics (MD) simulations using the Desmond module of the Schrodinger package on a GPU-enabled Linux computer. To simulate physiological conditions, the model was created in a TIP3P (transferable intermolecular potential-three point), orthorhombic water buffered solvation box of 10 Å dimensions and 0.15 M salt. The simulation model was then minimized and neutralized by adding sodium (Na^+^) and chloride (Cl^−^) ions, placed at least 20 Å from the ligands. The protein-ligand complexes were simulated for one thousand nanoseconds in an OPLS-2005 force field. At the end of the simulation period, the root mean square deviation (RMSD), root mean square fluctuation (RMSF), radius of gyration (Rg), Ligand RMSD, solvent accessible surface area (SASA), and intramolecular hydrogen bonds (intraHB) were computed and subsequently analyzed (Chow et al., 2008; Bergdorf et al., 2014; Release, 2014).

### 2.7 Molecular mechanics/generalized born and surface area solvation (MMGBSA) calculations

Molecular mechanics/generalized Born solvent area (MM/GBSA) calculations (Kollman et al., 2000; Massova and Kollman, 2000; Tsui and Case, 2000) were performed to estimate the relative binding energies of the selected ligands to CDK1. This method estimates the amount of free energy involved in interactions of the protein-ligand complex. The binding energy for 2000 independent snapshots of each ligand-protein complex was generated and average binding energies calculated. ΔG of each ligand was calculated following the given equation:

$$\Delta G_{\text{bind}}=\Delta G_{\text{RL}}\;–\;\left(\Delta G_{R}+\Delta G_{L}\right)$$ (1)

Where ΔG_RL_ represents the free energy of protein-ligand complex; ΔG_R_ is free energy of receptor, and ΔG_L_ the free energy of ligand.

The free binding energy establishes the binding affinity of ligands to a given protein. A more negative ΔG value indicates better binding of the ligand to the protein (Gilson et al., 1997). The free energy of each state was calculated, using FF14SB force field, by the given equations…

$$\Delta G_{\text{bind}}=\Delta E_{\text{gas}}+\Delta G_{\text{sol}}\;–T\Delta S$$ (2)

$$E_{\text{gas}}=E_{\mathrm{int}}+E_{\text{vdW}}+E_{\text{ele}}$$ (3)

$$G_{\text{sol}}=G_{\text{GB}}+G_{\text{SA}}$$ (4)

$$G_{\text{SA}}=ɣ\text{SASA}$$ (5)

Contribution of each residue to the total binding free energy was also obtained using MM/GBSA method in Amber 18.

## 3 Results

### 3.1 Analysis of the binding modes of co-lig and hit compounds in CDK1 active site

**FIGURE 2:**
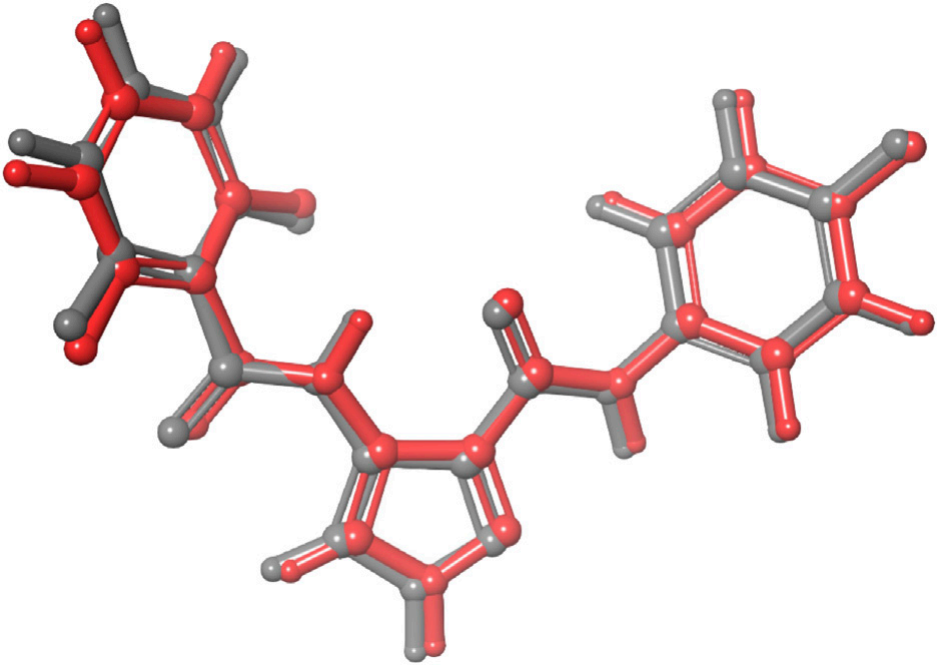
Validation of docking showing the co-crystallized ligand (in grey) and the re-docked pose (in red) with an RMSD score of 0.28 Å. The low RMSD value obtained demonstrates the efficacy of the docking protocol used.

**FIGURE 3:**
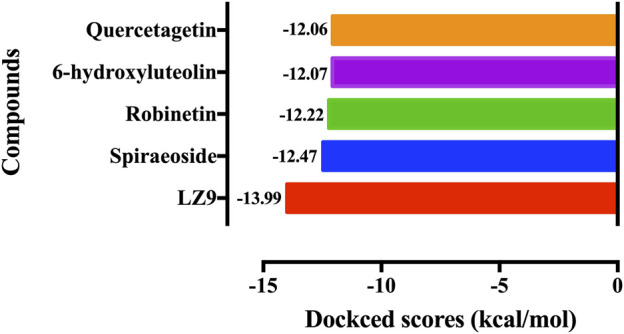
Molecular docking results of the top four scoring compounds: Spiraeoside, Robinetin, 6-hydroxyluteolin, Quercetagetin and the co-crystalized ligand (LZ9).

**FIGURE 4:**
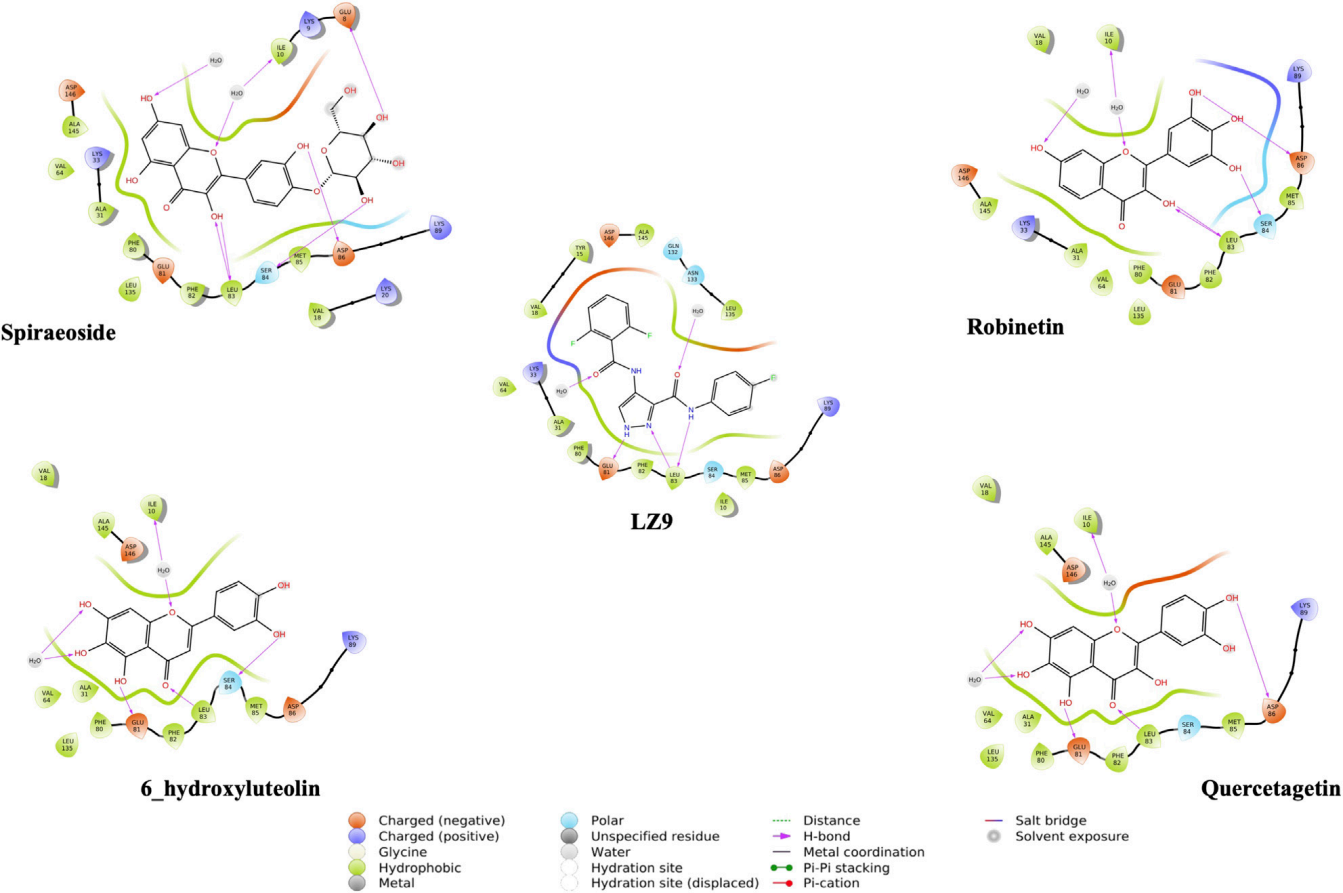
2D interaction diagrams of top scoring compounds in complex with CDK1 from molecular docking studies. Maestro 2D interaction diagram (Schrödinger) was used to generate the image.

**FIGURE 5:**
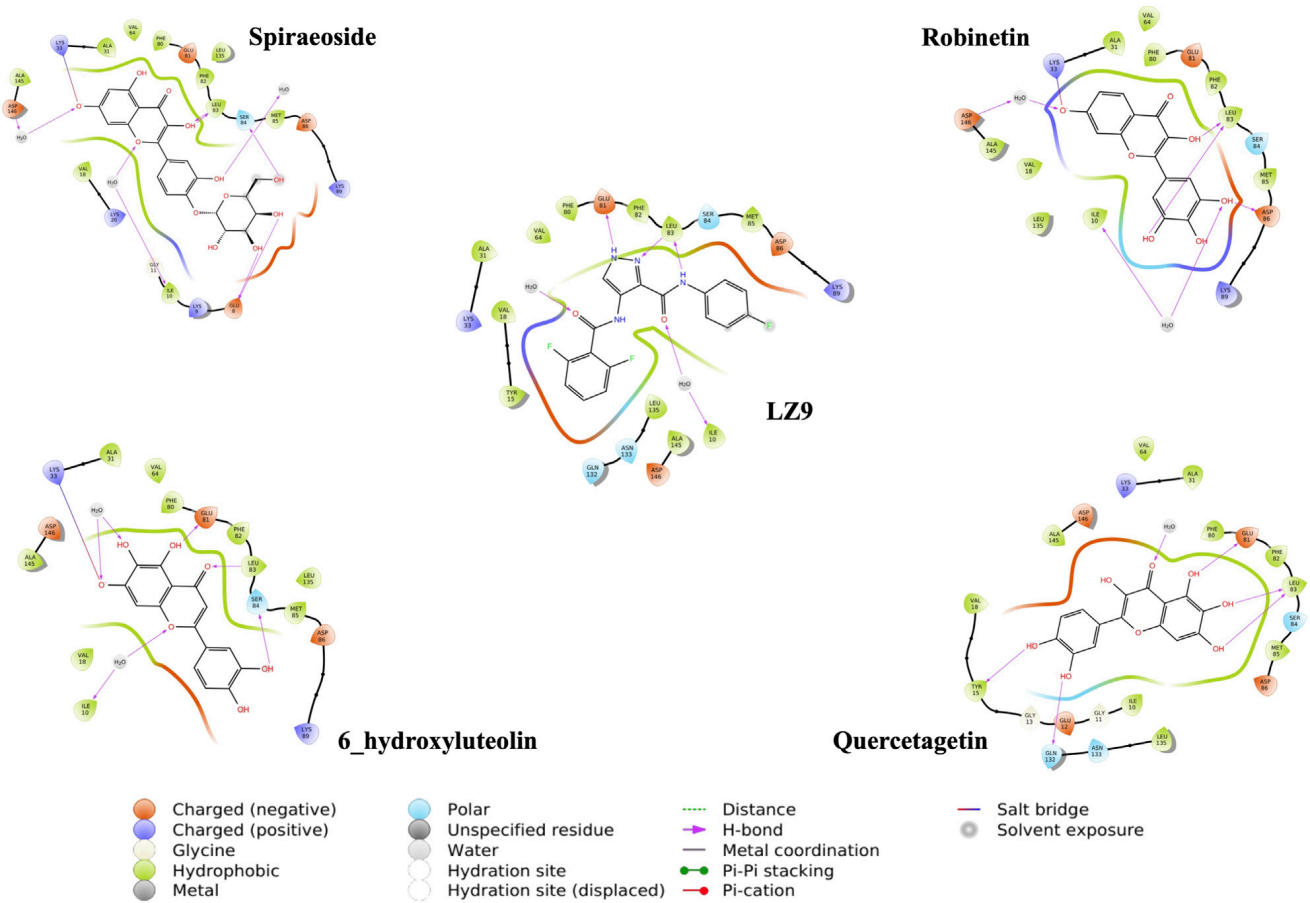
2D interaction diagrams of poses from Induced fit docking.

We performed molecular docking study of the phyto-compounds against CDK1 target protein. We selected the top four hits with the most favourable binding scores for subsequent studies. To validate the docking procedure, the co-crystallized ligand was re-docked into the binding pocket of CDK1. The RMSD between docked and native pose (Figure 2) is given as 0.28 Å. Figure 3 depicts the result from molecular docking studies of the four top-scoring compounds: Spiraeoside (−12.47 kcal/mol), Robinetin (−12.22 kcal/mol), 6-hydroxy luteolin (−12.07 kcal/mol), Quercetagetin (−12.06 kcal/mol), and the co-crystallized ligand, LZ9 (−13.99 kcal/mol). The 2D protein-ligand interaction diagrams (Figure 4) show the hydrogen and hydrophobic bond network and interactions shared between the compounds and CDK1. In this study, it was observed that all 4 hit compounds formed hydrogen bonds and hydrophobic interactions with the Ile10 residue of CDK1. Hydrogen bond interactions also existed between the compounds and CDK1 via Leu83, Ser84, and/or Gln 132 residues. Hydrophobic interactions were seen between all the compounds and Ile10, Val18, Ala31, Val64, Phe80, Phe82, Leu83, Met85, Leu135 and Ala145 residues of CDK1. LZ9 shared hydrophobic bonding with Tyr15, in addition to the aforementioned residues. Hydrophobic bonds observed from molecular docking were preserved during induced fit docking. Bond interactions for molecular docking, induced fit docking and molecular dynamics simulation are summarized in Table 1. The complexes formed from induced-fit docking are displayed in Figure 5.

**TABLE 1:** Bond interactions between ligands and CDK1 during molecular docking, induced fit docking and molecular dynamics simulation.

Compounds	Hydrogen bonds			π- π bonds
	MD	IFD	MDS	MDS
LZ9	Glu81, leu83	Ile10, Glu81, Leu83	Glu81, Leu83, Asp146	Tyr15
Spiraeoside	Glu8, Ile10, Leu83, Ser84, Asp86	Glu8, Ile10, Lys33 Leu83, Ser84, Asp146	—	—
Robinetin	Ile10, Leu83, Ser84, Asp86	Ile10, Lys33, Leu83, Asp86, Asp146	Thr14, Lys33, Val64, Glu81, Leu83, Arg158	Phe80
6_hydroxyluteolin	Ile10, Glu81, Leu83, Ser84	Ile10, Lys33, Glu81, Leu83, Ser84	Thr14, Lys33, Glu51, Leu83, Arg158, Phe147	—
Quercetagetin	Ile10, Glu81, leu83, asp86	Tyr15, Glu81, Leu83, Gln132	Lys33, Glu51, Leu83	—

MD, molecular docking; IFD, induced fit docking; MDS, molecular dynamics simulation.

### 3.2 *In silico* ADME predictions

Table 2 gives details of ADME and toxicity predictions. Physicochemical descriptors, ADME (Absorption, Distribution, Metabolism, and Excretion) parameters, and pharmacokinetic properties of the compounds, except spiraeoside, were predicted to be within the permissible parameter ranges.

**TABLE 2:** Predicted physicochemical descriptors, ADME parameters, pharmacokinetic properties, druglike nature, medicinal chemistry friendliness and toxicity of compounds.

	LZ9	Spiraeoside	Robinetin	6-Hydroxyluteolin	Quercetagetin
Physicochemical properties					
Molecular weight (g/mol)	360.29	464.38	302.24	302.24	318.24
Heavy atoms	26	33	22	22	23
Rotatable bonds	6	4	1	1	1
Hydrogen bond acceptor	6	12	7	7	8
Hydrogen bond donor	3	8	5	5	6
TPSA (Å^2^)	86.88	210.51	131.36	131.36	151.59
Lipophilicity					
Log *P*_o/w_ (XLOGP3)	3.07	1.31	1.61	2.17	1.18
Log *P*_o/w_ (WLOGP)	4.21	−0.54	1.99	1.99	1.69
Log *P*_o/w_ (MLOGP)	2.64	−2.59	−0.56	−0.56	−1.08
Consensus Log P_o/w_	3.03	−0.19	1.12	1.42	0.92
Water solubility					
LogS (ESOL)	−4.10	−3.64	−3.20	−3.55	−3.01
Solubility (mg/mL)	2.89 e−02	1.07e-01	1.91e-01	8.46e-02	3.14e-01
Class	Moderately soluble	Soluble	Soluble	Soluble	Soluble
Pharmacokinetics					
GI absorption	High	Low	High	High	Low
BBB permeant	No	No	No	No	No
P-gp substrate	No	Yes	No	No	No
CYP1A2 inhibitor	Yes	No	Yes	Yes	Yes
CYP2C19 inhibitor	No	No	No	No	No
CYP2C9 inhibitor	No	No	No	No	No
CYP2D6 inhibitor	Yes	No	Yes	Yes	No
CYP3A4 inhibitor	No	No	Yes	Yes	Yes
Druglikeness					
Lipinski	Yes (0 violation)	No (2 violations)	Yes (0 violation)	Yes (0 violation)	Yes (1 violation)
Ghose	Yes	No	Yes	Yes	Yes
Veber	Yes	No	Yes	Yes	No (1 violation)
Bioavailability score	0.55	0.17	0.55	0.55	0.55
Leadlikeness	No	No	Yes	Yes	Yes
Toxicity					
Predicted LD 50 (mg/kg)	1,000	5,000	159	3,919	159
Toxicity Class	4	5	3	5	3
Hepatotoxicity (probability score)	Active (0.65)	Inactive (0.82)	Inactive (0.69)	Inactive (0.69)	Inactive (0.69)
Carcinogenicity (probability score)	Active (0.53)	Inactive (0.85)	Active (0.68)	Active (0.68)	Active (0.68)
Mutagenicity (probability score)	Inactive (0.71)	Inactive (0.76)	Active (0.51)	Active (0.51)	Active (0.51)

From toxicity prediction, spiraeoside was predicted to be inactive for all parameters. LZ9 was predicted to be hepatotoxic and carcinogenic, but not mutagenic. Robinetin, 6-hydroxyluteolin, and quercetagetin were predicted to be inactive for hepatotoxicity, while results for carcinogenicity and mutagenicity were predicted to be active. Their probability scores are shown in brackets.

### 3.3 RMSD analysis of CDK1 and hit compounds

**FIGURE 6:**
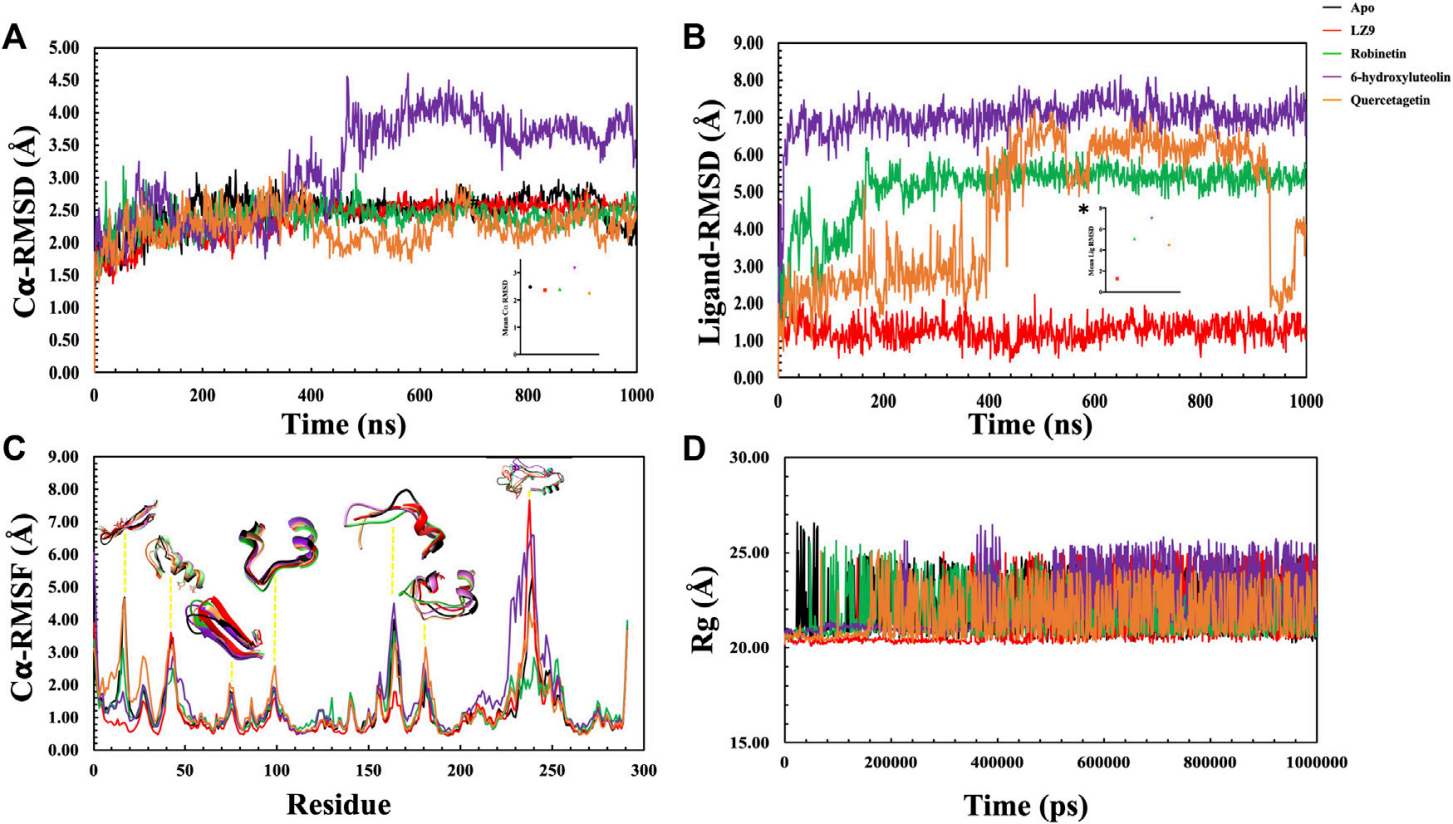
Molecular dynamics simulation trajectories of CDK1 in presence of ligands and absence of ligand (Apo) as a function of 1,000 ns simulation time. **(A)** Root Mean Square Deviation (RMSD) of Cα atoms backbone. Insert, # shows the mean Cα RMSD values. **(B)** Root Mean Square Deviation of the ligands with respect to CDK1 protein. The plot displays the RMSD of the ligands after measuring the RMSD of the heavy atoms in the ligand and aligning the protein-ligand complex on the reference protein backbone. If the reported values are noticeably greater than the protein’s RMSD, the ligand has probably diffused away from its initial binding site. Insert, * shows the mean RMSD values. **(C)**: Root Mean Square Fluctuation (RMSF) per residue over time. **(D)**: Radius of gyration for the Apo state and CDK1-ligand complexes.

The protein backbone RMSD (C𝛂) & ligand heavy atoms RMSD over the course of simulation time were plotted. The average values of each system were also calculated. The RMSD depicts the dynamic behavior and stability of the complexes over the simulation period and estimates the average change in atom displacement for a given frame relative to a reference frame. All protein frames were first aligned on the reference frame backbone before determining the RMSD based on the atom selection. RMSD fluctuations give insight to the stability of a ligand within the binding pocket of a protein. A gradual increase in RMSD values was observed in the first 200 ns of the apo protein system, after which it stabilised and had an average value of 2.48 Å (Figure 6A). The CDK1-LZ9 system fluctuated for the first 400 ns, after which it stabilised and had an average of 2.36 Å. CDK1-robinetin stystem stabilised after 200 ns with an average value of 2.40 Å, while the CDK1-quercetagetin system fluctuated more, however, had an average value of 2.24 Å. The most fluctuations were observed in the CDK1-6-hydroxyluteolin system which also had the highest mean value of 3.17 Å. From the ligand RMSD plot (Figure 6B), the ligands displayed quite stable trends over the simulation period, except for quercetagetin, which fluctuated significantly after 400 ns, even though it had an average RMSD value of 4.48 Å. LZ9 had an average RMSD value of 1.27 Å, robinetin, 5.10 Å and 6-hydroxyluteolin, 7.00 Å.

### 3.4 RMSF analysis of CDK1 and hit compounds

In general, secondary structure components of a protein like alpha helices and beta strands are more rigid than the unstructured part and fluctuate less than loop regions during molecular dynamics simulation runs. Analysis of the RMSF plots (Figure 6C), show that all the systems displayed similar patterns. Notable distinctions include quercetagetin with the highest peak value at residue 100, robinetin also displayed highest peak at residue 130, whereas LZ9 had highest peak value at residue 240. LZ9 had the least peak at residue 165 while robinetin had the least value between residues 230 and 240 where other system had highest peaks. A comparative 3D representation of the crucial residue regions of the systems which exhibited higher fluctuations has equally been presented.

### 3.5 Radius of gyration, solvent accessible surface area and intra-hydrogen bond analysis

**FIGURE 7:**
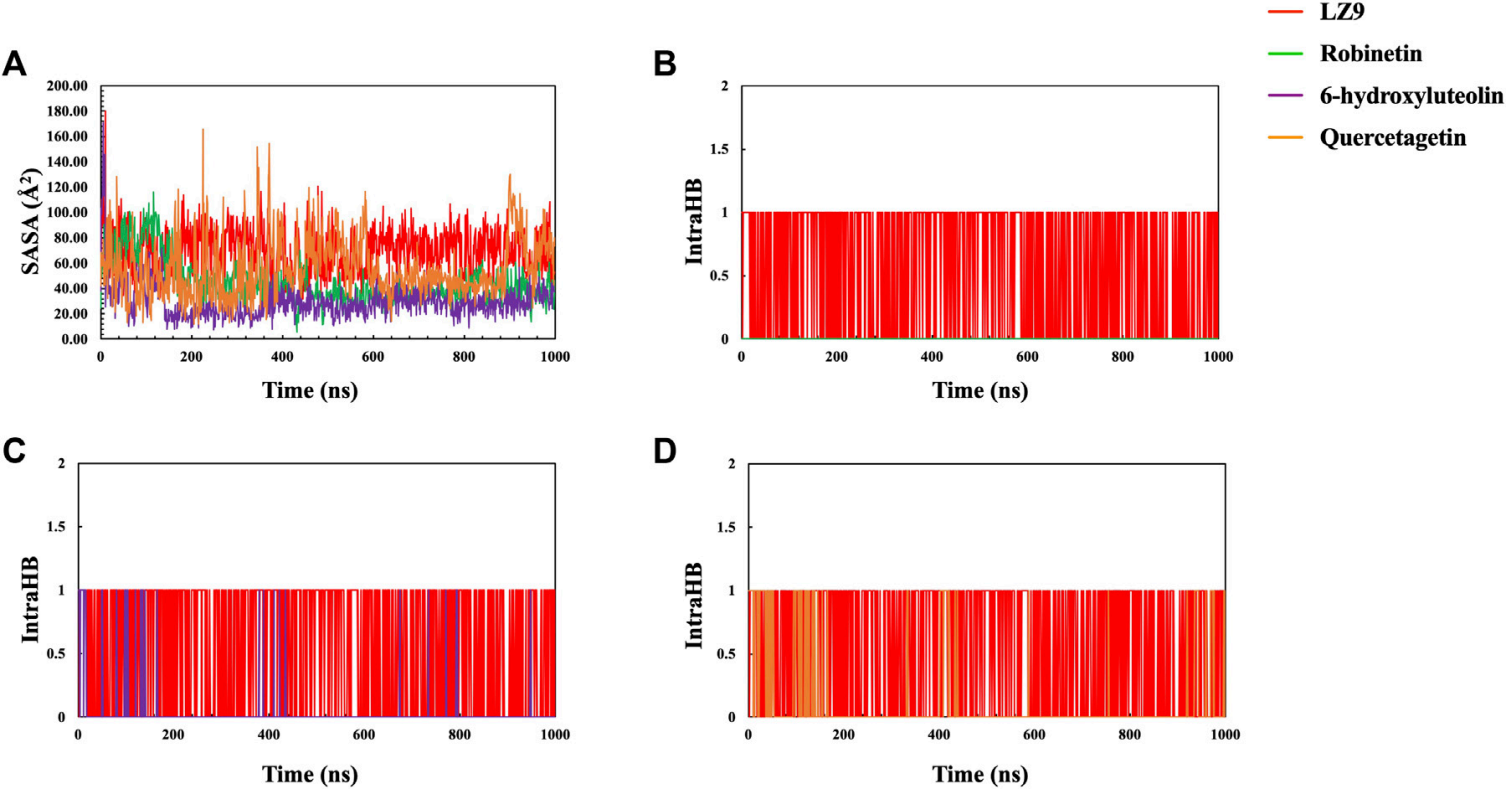
**(A)** Solvent Accessible Surface Area (SASA) plot. Intramolecular Hydrogen Bonds (intraHB) plot showing the number of internal hydrogen bonds within the ligand molecules—Robinetin **(B)**, 6-hydroxyluteolin **(C)**, Quercetagetin **(D)**, compared with LZ9.

The radius of gyration for all systems is shown in Figure 6D. When comparing the plots of the apo form to the CDK1 bound states, there is no apparent alteration to the global protein compactness in response to ligand binding. The solvent-accessible surface area describes the surface area of a molecule accessible to water. In addition to examining the impacts on the molecule, it expresses the degree to which a simulated protein interacts with solvent molecules. Evaluating the SASA value is an important way to estimate conformational changes caused by complex interactions. This is essential for characterizing protein-ligand interactions and understanding structural changes during simulations. Solvent Accessible Surface Area (SASA) plots (Figure 7A) show that LZ9 and quercetagetin have high SASA values, while robinetin and 6-hydroxyluteolin have lower SASA values. Intramolecular hydrogen bonds show the number of internal hydrogen bonds (HB) within a ligand molecule and give insight into the behavior of ligands in the active site of a protein. They can also stabilize conformations that are conducive to protein binding, thus increasing the affinity for the receptor. Intra-hydrogen bond (intraHB) plots (Figures 7B–D) reveal that LZ9 had intraHB interactions almost throughout the simulation time, 6-hydroxyluteolin and quercetagetin had more intraHB interactions at the beginning of the simulation, while robinetin maintained a value of zero, that is zero intraHB interactions throughout the simulation time.

### 3.6 Protein-ligand contacts

**FIGURE 8:**
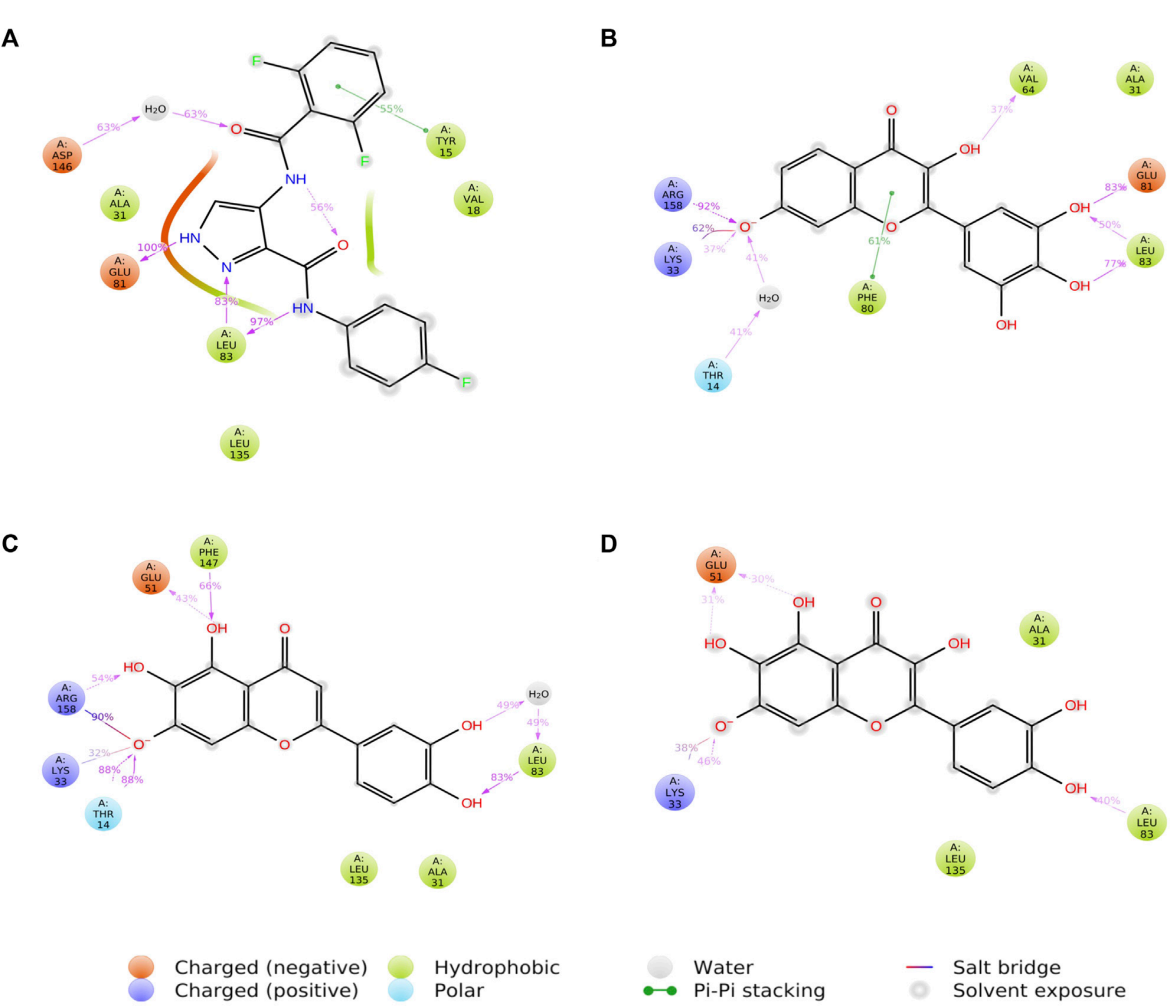
A schematic of detailed ligand atom interactions of LZ9 **(A)**, Robinetin **(B)**, 6-hydroxyluteolin **(C)** and Quercetagetin **(D)** with the protein residues of CDK1. Interactions that occur more than 30.0% of the simulation time in the selected trajectory (0 through 1000 ns) are shown.

**FIGURE 9:**
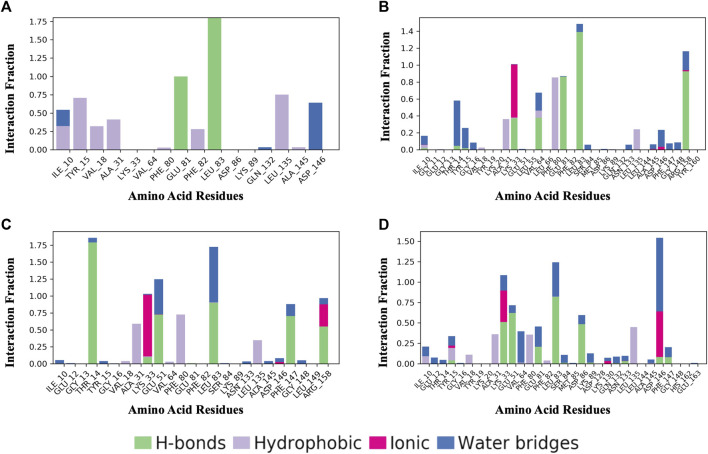
Protein-Ligand Contacts. **(A)** LZ9. **(B)** Robinetin. **(C)** 6-hydroxyluteolin. **(D)** Quercetagetin. Contact interactions can be categorized by type and summarized, as shown in the plot. Protein-ligand contacts are categorized into four types: Hydrogen bonds, Hydrophobic, Ionic and Water Bridges. The stacked bar charts are normalized over the course of the trajectory: for example, a value of 0.7 suggests that 70% of the simulation time the specific interaction is maintained. Values over 1.0 are possible as some protein residue may make multiple contacts of same subtype with the ligand.

Figure 8 shows a schematic of the detailed ligand atom interactions with the protein residues. The interactions in this trajectory that occur for more than 30% of the simulation period are displayed. LZ9 and robinetin make pi-pi contacts with Tyr15 and Phe80 for more than 50% of the time. All the ligands made contact with Leu83 via hydrogen bonds; LZ9 and robinetin form hydrogen bonds with Glu81, while 6-hydroxy luteolin6-hydroxy luteolin and quercetagetin form hydrogen bonds with Glu51. Robinetin, 6-hydroxyluteolin, and quercetagetin form salt bridges with Lys33. All the ligands show relatively high solvent exposure. Protein-ligand contacts as illustrated by bar charts (Figure 9) during the simulation, show that hydrogen bonds, hydrophobic interactions, and water bridges were predominant in LZ9. The other ligands had ionic interactions in addition to the aforementioned. All compounds exhibited strong hydrogen bonding on Leu83 and hydrophobic interactions on Leu135. They also showed a water bridge on residue Asp146. Only the lead compounds showed ionic bonds at residue Lys33.

### 3.7 Potential energy

**FIGURE 10:**
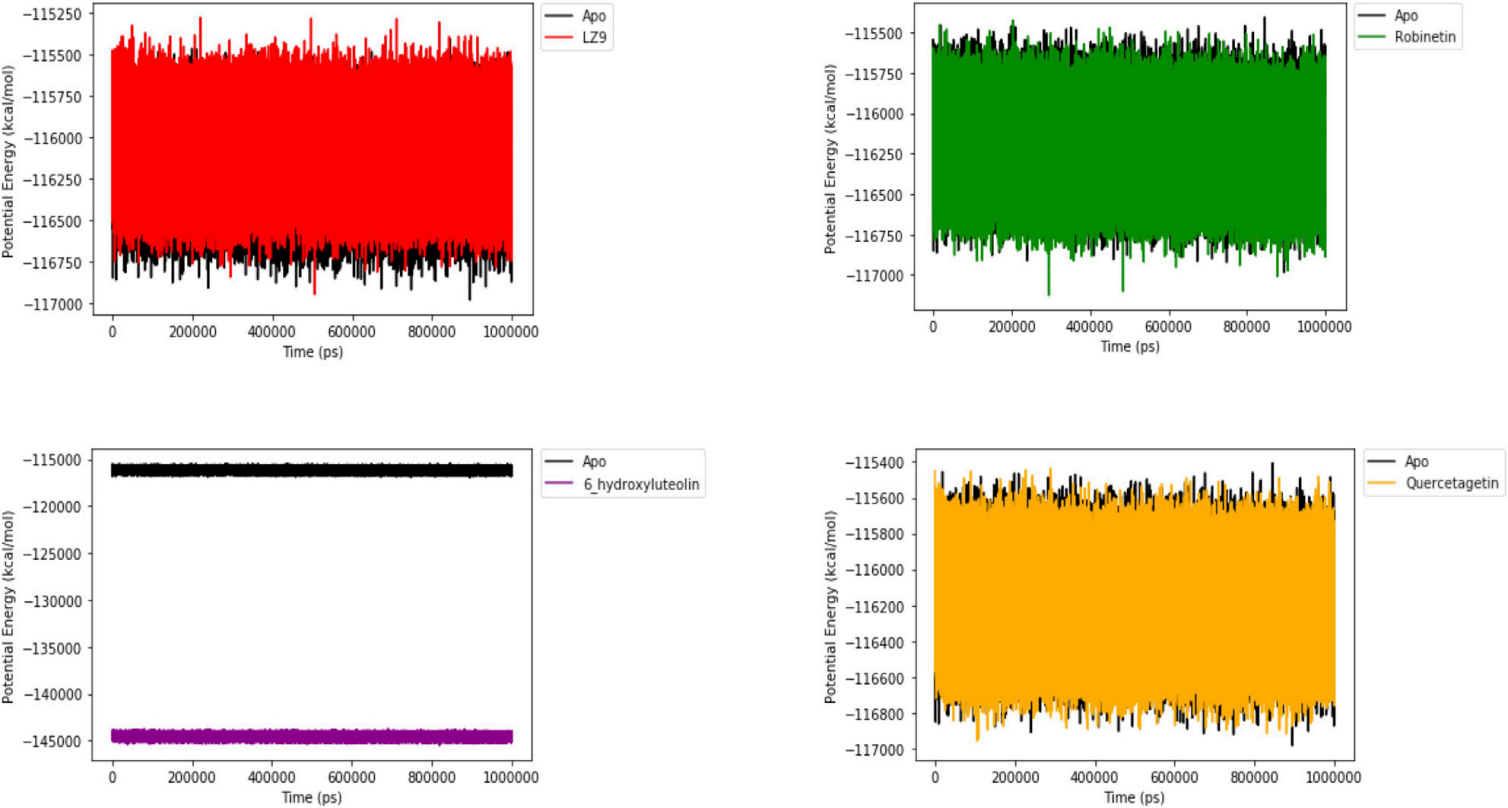
Potential energy plot of 1,000 ns simulation time. The plots show the energies of the systems in apo and bound states.

Figure 10 shows the potential energy plot of the systems in the apo state and in complex with the ligands. When bound to LZ9, robinetin and quercetagetin, we observe energies in the same range as that of the apo state; −115,500 to −117,000 kcal/mol. On the other hand, when bound to 6-hydroxyluteolin, a lower energy state of −144,000 kcal/mol is observed.

### 3.8 Free binding energy

**FIGURE 11:**
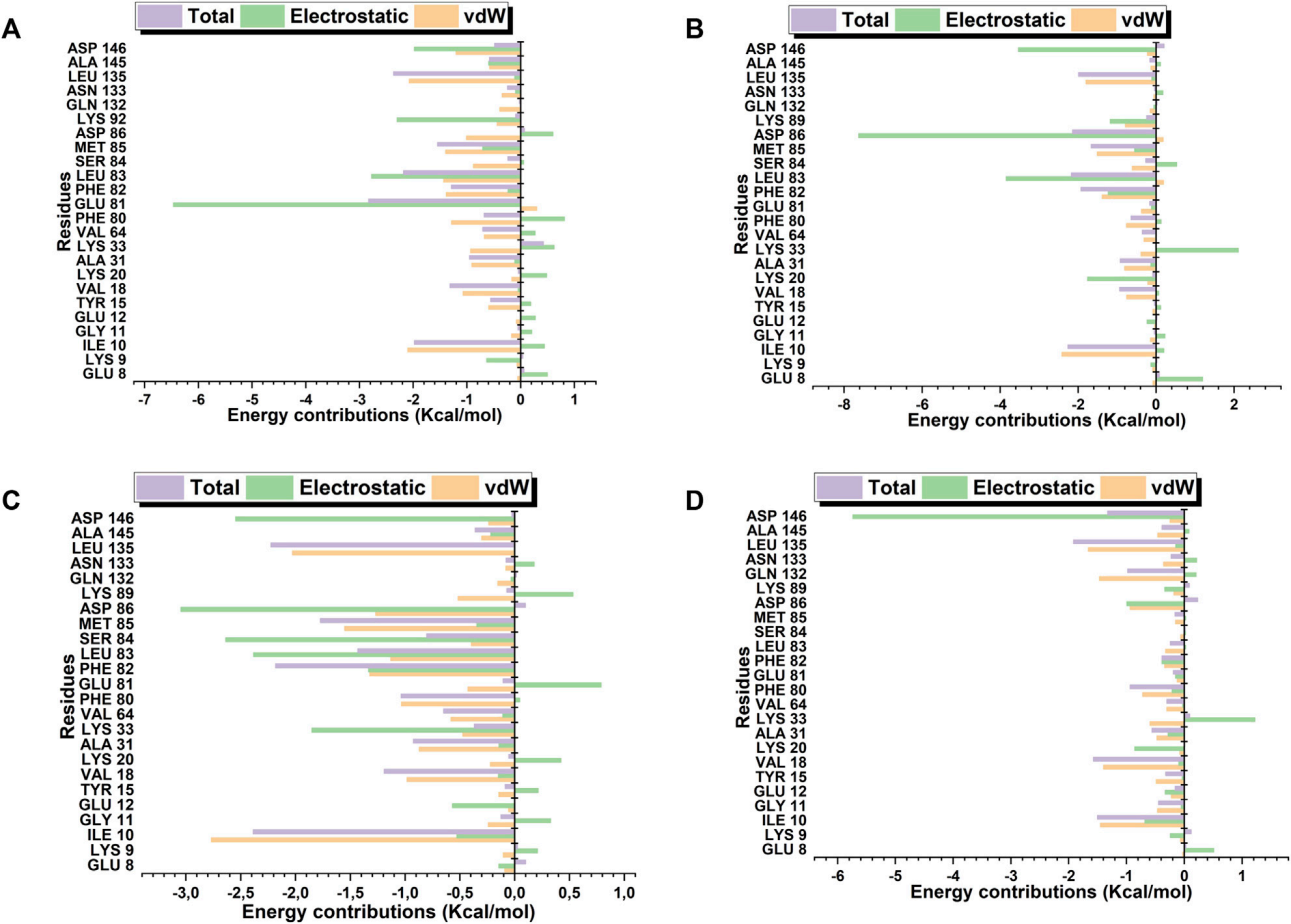
Active site residues and the resultant energy contributions in stabilizing the complexes formed between CDK1 and LZ9 **(A)**; robinetin **(B)**; 6_hydroxyluteolin **(C)**; and Quercetagetin **(D)**.

Robinetin and 6-hydroxyluteolin exhibited good ΔG values of −30.40 kcal/mol and −30.63 kcal/mol, respectively (Table 3). The co-lig, LZ9, had a value of −34.96 kcal/mol. Quercetagetin had a binding energy of −18.52 kcal/mol. Energy contributions of active site residues responsible for stabilizing the CDK1-ligand complexes are shown in Figure 11. Worthy of note is Glu81 as the residue showing the highest energy contribution in the CDK1-LZ9 complex, while Asp86 is the residue showing highest binding energy contribution to the CDK1-robinetin and CDK1-6-hydroxyluteolin complexes. The CDK1-quercetagetin system has Asp146 as the active site residue contributing the highest energy. Visual inspection of the figure shows that Van der Waals forces contribute more to the interaction between CDKI-LZ9 and CDK1-6-hydroxyluteolin complexes. Robinetin has more electrostatic energy contributing to the stability of the complex it forms with CDK1. Quercetagetin forms weak interaction with CDK1 as a result of the overall low Van der Waals forces and electrostatic energy.

**TABLE 3:** Energy component calculations obtained by MM/GBSA method.

Compounds	Energy components (kcal/mol)				
	ΔE_vdw_	ΔE_ele_	ΔG_gas_	ΔG_sol_	ΔG_bind_
LZ9	−41.88 ± 2.97	−22.02 ± 3.62	−63.90 ± 4.39	28.95 ± 2.98	−34.96 ± 2.90
Robinetin	−28.32 ± 3.22	−36.89 ± 7.32	−65.21 ± 6.41	34.81 ± 5.61	−30.40 ± 2.66
6-hydroxyluteolin	−37.20 ± 2.32	−28.94 ± 7.81	−66.14 ± 7.60	35.51 ± 5.17	−30.63 ± 3.78
Quercetagetin	−29.00 ± 3.043	−15.95 ± 10.06	−44.95 ± 10.04	26.43 ± 6.44	−18.52 ± 5.17

ΔE_vdw_, Van der Waals energy; ΔE_ele_, Electrostatic energy; ΔG_gas_, Gas phase energy; ΔG_sol_, Solvation energy; ΔG_bind_, Total binding energy.

## 4 Discussion

The prevalence of colorectal cancer is fast becoming a public health concern (Holowatyj et al., 2020). Development of new pharmacologically therapeutic anti-cancer drug agents has been hampered by the limited molecular understanding of the oncogenetic initiation and the interaction of biochemical mechanisms/factors, although traditional chemotherapy options have produced impressive results in the treatment of CRC with the possibility of relapses.

Natural products are a valuable source of novel therapeutic leads given that they contain a wide variety of chemical components (Cragg and Newman, 2013; Newman and Cragg, 2016). They have been essential in the development of new drugs, particularly for the treatment of cancerous disorders (Atanasov et al., 2015; Harvey et al., 2015). The compounds identified from this study were found to be in the class of flavonoids. There is growing evidence that flavonoids may be useful in the prevention and treatment of cancer and they are also reported to have a variety of anti-cancer effects, including inducing apoptosis in cancer cells, inhibiting cell proliferation, and may enhance chemotherapy (Abotaleb et al., 2018). Flavonoids have also been reported to play a potential role in colorectal cancer therapies (Amado et al., 2014).

Given the importance of cyclin-dependent kinases (CDK) in cancer, targeting CDKs is a viable strategy for combating the cancer menace (Sofi et al., 2022a). Because of the significant role it plays in one of the phases of cell cycle control, which is a crucial one for the growth of tumor cells, specifically targeting CDK1 became a strategic focus for optimized treatment. The drug discovery pipeline greatly benefits from the use of computational screening techniques (Choudhury et al., 2022). In recent years, computer-aided drug development methods have been widely used to find lead compounds with therapeutic value. These technologies include molecular docking, *in silico* pharmacokinetic profiling, and molecular dynamics simulations (MDS) (Wan, 2013; Salmaso and Moro, 2018). MDS are anticipated to become more significant as new pharmacological therapies are developed (Durrant and McCammon, 2011). Several studies now use these computational methods to quickly identify potential inhibitors of key proteins in disease progression as treatment options (Choudhary et al., 2020; Sofi et al., 2022a; Rathod et al., 2022; Castrosanto et al., 2023) others have identified experimentally validated leads (Kalani et al., 2013; Yilmaz Goler et al., 2022). Here, we used these computational techniques to screen phyto-compounds and identify possible inhibitors of CDK1.

Molecular docking is a computational method used to predict the preferred binding orientation of a ligand to a receptor. The software algorithm used predicts possible binding orientations and provides scores ranked according to the predicted binding affinity between the receptor-ligand complex (Huang and Zou, 2010; Salmaso and Moro, 2018). To better understand the binding dynamics of the four compounds selected in this study to CDK1, they were subjected to molecular dynamics simulations (MDS). Prior to running simulation studies, we carried out induced fit docking (IFD) (Achilonu et al., 2020). Induced fit docking is a more realistic model of how proteins and ligands interact in nature as it considers the flexibility of the protein and ligand, allowing them to move and rotate during the docking process, resulting in more accurate predictions of the binding of ligands. For traditional rigid docking, the proteins and ligands are treated as rigid bodies, and the docking algorithm attempts to find the best orientation of the ligand in the protein’s binding site. However, in reality, proteins are flexible molecules, which can undergo conformational changes upon ligand binding. Both minor backbone relaxations and substantial side-chain conformational changes in the receptor structure are accounted for by the induced-fit docking (IFD) technique (Sherman et al., 2006; Xu and Lill, 2013).

All systems ran through the complete one thousand nanoseconds duration during MDS, except spiraeoside, which ran for only 700 ns? It could be that due to conformational changes in the protein, spiraeoside left the binding site of the protein during the simulation run. For this reason, the post-simulation analysis could not be completed, consequently, results for spiraeoside were excluded.

It is important to pay attention to the protein’s RMSD value to properly infer structural changes occurring in a protein which could affect ligand binding. C𝛂-RMSD demonstrated a stable structural conformation across the course of the simulation, showing that it was balanced. The low RMSD values of LZ9, robinetin and quercetagetin indicate low fluctuations and suggests that the system maintained stability during simulation and the protein did not undergo significant structural changes as a result of binding of the ligands. The higher RMSD value of 6-hydroxyluteolin system might suggest that the binding of this ligand might have perturbed the structure of the CDK1 protein.

Ligand RMSD provides insight into how stably the bound ligand sits within the protein binding pocket. The Ligand RMSD results show that LZ9, robinetin, and 6-hydroxyluteolin did not move much, therefore forming a stable complex with CDK1, unlike quercetagetin which had a lot of fluctuations. The fluctuations observed with quercetagetin after 400 ns tell us that it is not tightly bound to the protein and has possibly diffused away from the binding pocket, so it forms an unstable protein-ligand complex and is less likely to perform its intended function.

RMSF measures the structural flexibility of a protein and characterizes local perturbations along a protein chain (Cob-Calan et al., 2019). It also provides information on how each amino acid residue is changing relative to its reference position during simulation (Sarkar and Sen, 2022). Here, we analysed the RMSF of the CDK1 protein in the apo and bound states. Peaks represent regions of the protein where the simulation-induced fluctuations were greatest (Tripathi et al., 2022). Illustrative 3D images of the peaks have been presented to clearly elucidate the silent fluctuation dynamics depicted by the represented peaks. The 3D images reveal a comparative conformational variation exhibited by the various system due to the perturbations created by the MD simulation. The 3D images have been color-coded to tally with their respective corresponding RMSF trajectories. The spikes observed from RMSF results may have been caused by increased flexibility within the protein’s loop regions. It may be inferred from the RMSF result that CDK1 has a great deal of flexibility to accommodate the ligand at the binding pocket.

The radius of gyration measures how compact a protein structure is and is equivalent to its principal moment of inertia, describing the common separation of each scattered element from the molecule’s mass center (Lobanov et al., 2008). During simulation, a more rigid structure is represented by lower Rg values (Jiang et al., 2019). Since not much difference was observed between the Apo and bound states, we can say that the binding of the ligands did not interfere with the compact structure of CDK1. In this study, the Rg, RMSD, and RMSF were correlated, and this relationship shed light on how complex compressibility affected the variations in complex RMSD and the residual fluctuations seen in RMSF. No noticeable changes are observed in the bound and unbound states of CDK1, suggesting that the protein structure retained its compact conformation. Our results indicate that the surface area of LZ9 and quercetagetin have higher exposure to water than robinetin and 6-hydroxyluteolin. This result is in tandem with that observed from the ligand-atom interaction diagram.

Hydrogen and hydrophobic bonds play key roles in protein-ligand interactions. Crucial hydrophobic and/or hydrogen bonds, including Ile10, Phe80, Leu83, and Ser84 between CDK1 and the lead compounds that were first seen after molecular docking were maintained during MDS. Phe80 has previously been described as a hydrophobic checkpoint enabling water molecules to gain access to the catalytic portion of the ATP catalytic domain for an additional hydrogen bond (McGrath et al., 2005). Robinetin is seen to form a pi-pi bond with Phe80 during simulation run, which may further account for its strong inhibitory properties. 6-hydroxyluteolin also forms a bond with Thr14 for 88% of the simulation time. Inhibition of Thr14 by small molecule compounds has been proposed as a promising option for the regulation of the G2/M transition of the cell cycle (Schmidt et al., 2017). Inhibition of CDK1 due to the phosphorylation of the kinase on Tyr15 residue by flavonoids (Casagrande and Darbon, 2001) as well as induction of G_2_/M phase arrest and apoptosis by the flavonoid tamarixetin on human leukemia cells (Nicolini et al., 2014) have previously been reported.

The hydrophobic and hydrogen bonds formed between the compounds and CDK1 may play a vital role in stabilizing the complexes for the compounds to exert their inhibitory efficacies (Zhang et al., 2011). Ionic bonds observed on only the lead compounds might also play a significant role in their inhibition of CDK1. Interestingly, the lead compounds are all of the flavonoid class; another study by ((Navarro-Retamal and Caballero, 2016)) also gave insight into flavonoids as CDK1 inhibitors. Natural flavonoids such as Luteolin and Daidzein have also been found to inhibit CDK1 (Ravishankar et al., 2013).

Higher free energy values in fact correlate with more preferable binding affinities of a ligand to a protein. Robinetin, which had good binding energy to CDK1, as revealed by the ΔG values, might have been a result of higher electrostatic and van der Waals energy contributions. Interactions between robinetin and Asp86 residue which was observed from molecular docking and induced fit docking results was maintained after the MM/GBSA run. We can say this interaction might be a key factor to stablizing the CDK1-robinetin complex. Energetic analysis reveals that van der Waals interaction and non-polar contributions are favorable in the formation of complexes and amine group of the ligand, which plays a crucial role for binding (Tripathi et al., 2012; He et al., 2023). Potential energy (PE) and RMSD are both vital indicators of protein stability. In previous work done by Yuan et al. (2012) to ascertain if the structures were stabilized, MD simulation was examined using PE and RMSD from the original model structure (Coutsias et al., 2004) The potential energy plot for 6-hydroxyluteolin is a reflection of the trajectory pattern exhibited by the Cα-RMSD plot of 6-hydroxyluteolin system between 230 ns and 420 ns, where a perturbation on the 6-hydroxyluteolin system seems to have created conformational alteration within the system, making it assume a new/different trajectory. The new structural conformational deviation of 6-hydroxyluteolin relative to other systems could presumably have produced the obtained PE.

The physicochemical properties of a compound are important parameters in the pipeline of drug development as they directly affect the absorption, distribution, metabolism, excretion, and toxicity profile (ADMET) of a drug candidate (Manallack et al., 2013). The drug-like and ADMET predictions are used in identifying properties of a ligand suitable for formulation into drugs. Even though all the compounds are adequately hydrophilic and lipophilic, robinetin and 6-hydroxyluteolin showed high intestinal absorption. This implies that the compounds are capable of achieving pharmaceutical and therapeutic bioavailability at the intestinal linings and target cells in the colon. This is further corroborated by their bioavailability scores. A study on natural compounds as inhibitors of CDK1 by Saikat (Saikat, 2021) also reported similar ADME properties.

Lipinski’s Rule of five is used to assess and predict the drug-likeness of compounds based on oral absorption, membrane permeability and bioavailability (Lipinski et al., 1997). Although, parenteral administration may be applicable in most cases of cancer treatment, oral administration is a valuable treatment option for cancer patients as it is more convenient and reduces the frequency of their clinic visits or venipuncture. In recent times, oral anti-cancer drugs such as capecitabine, topotecan and vinorelbin are successfully used in the treatment of a variety of tumours like colon, breast, ovarian or lung cancer (Colomer et al., 2010). With the advent of targeted deliveries, most of the drug molecules approved as oral medications for cancer treatment including hormone therapies such as, dasatinib, sorafenib, imatinib, lapatinib and abiraterone have resulted in good clinical outcomes (Gralow et al., 2008; Rehman and Rosenberg, 2012).

Despite the fact that natural products frequently violate these rules due to their complexity, they still serve as reliable sources for novel drug discovery and development (Doak et al., 2014). Some anti-cancer agents such as vincritine, irinotecan, paclitaxel and etoposide, widely used for decades in cancer treatments originate from natural compounds (Huang et al., 2021).

The ProTox–II server is a web-based tool that predicts the potential toxicity of a given small molecule compound on various organs and systems in the body. The toxicity is predicted using a machine learning model that has been trained on a sizable dataset of toxic and non-toxic molecules. The toxicity prediction and probability score are considered important parameters for predicting a compound’s toxicity (Han et al., 2019). The probability score can be used to evaluate the prediction’s level of confidence. A forecast with a high probability score is more certain to occur, whereas one with a low probability value may not. Additionally, if the organ toxicity and/or toxicity end points of a compound are classified as active, it suggests that the compound may have harmful effects if it is ingested, inhaled, or otherwise exposed to the body. Conversely, “inactive” could mean that the compound has not been predicted to have a toxic effect. Early identification of toxicity in the drug development steps is critical in reducing late-stage attrition rates (Roberts et al., 2014; Segall and Barber, 2014). The predicted toxicity class exhibited by the compounds indicates that they can be administered within the accepted safety doses. Although the test ligands are predicted to be non-hepatotoxic, lead optimization may be required to modify the other toxicity endpoints.

## 5 Conclusion and next steps

In this study, computational techniques including molecular docking, induced fit docking, molecular dynamics simulation and MM/GBSA were used to study the potential inhibitory activities of natural compounds against CDK1. The findings provided insight into molecular interactions of significant residues involved in inhibitory CDK1 activities through molecular docking and molecular dynamics simulation results. The current investigation of small molecule inhibitors in the activity of the CDK1 explored viable options for the roles of Robinetin and 6-hydroxyluteolin in the pathogenesis of CRC. This research, however, provides a basis for further examination of potential phytochemicals for medical intervention of several cancers. This study aimed to identify potential small molecule inhibitors of CDK1 hence the use of molecular modeling approaches was deployed to highlight compounds of strong binding energies, good spatial amino acid residue arrangement, and better pharmacokinetic properties among others. The MD simulations showed that the hit compounds stably interacted with the CDK1 enzyme and maintained firm positions within the binding pocket of the enzyme over the simulation timeframe. The findings of this study should undergo experimental validation to confirm the interaction between the top ligands identified in our research and the target protein. Additionally, the ADME/Tox properties that were predicted using computational models need to be verified through experimental testing.

## Data Availability

Publicly available datasets were analyzed in this study. This data can be found here: NCBI PDB.

## References

[B1] Abotaleb M. Samuel S. M. Varghese E. Varghese S. Kubatka P. Liskova A. (2018). Flavonoids in cancer and apoptosis. Cancers 11 (1), 28. 10.3390/cancers11010028 30597838 PMC6357032

[B2] Achilonu I. Iwuchukwu E. A. Achilonu O. J. Fernandes M. A. Sayed Y. (2020). Targeting the SARS-CoV-2 main protease using FDA-approved Isavuconazonium, a P2–P3 α-ketoamide derivative and Pentagastrin: an in-silico drug discovery approach. J. Mol. Graph. Model. 101, 107730. 10.1016/j.jmgm.2020.107730 32920239 PMC7462840

[B3] Adeoti M. L. Oguntola S. A. Olugbenga-Bello A. I. Oladimeji O. J. Jegede S. O. (2016). Colorectal cancer: knowledge and risk factors among adults in a sub urban Nigeria community. J. Med. Sci. Clin. Res. 4 (9), 1247. 10.18535/jmscr/v4i9.28

[B4] Adhikari D. Zheng W. Shen Y. Gorre N. Ning Y. Halet G. (2012). Cdk1, but not Cdk2, is the sole Cdk that is essential and sufficient to drive resumption of meiosis in mouse oocytes. Hum. Mol. Genet. 21 (11), 2476–2484. 10.1093/hmg/dds061 22367880

[B5] Amado N. G. Predes D. Moreno M. M. Carvalho I. O. Mendes F. A. Abreu J. G. (2014). Flavonoids and wnt/β-catenin signaling: potential role in colorectal cancer therapies. Int. J. Mol. Sci. 15 (7), 12094–12106. 10.3390/ijms150712094 25007066 PMC4139831

[B6] Atanasov A. G. Waltenberger B. Pferschy-Wenzig E. M. Linder T. Wawrosch C. Uhrin P. (2015). Discovery and resupply of pharmacologically active plant-derived natural products: a review. Biotechnol. Adv. 33 (8), 1582–1614. 10.1016/j.biotechadv.2015.08.001 26281720 PMC4748402

[B7] Banerjee P. Eckert A. O. Schrey A. K. Preissner R. (2018). ProTox-II: a webserver for the prediction of toxicity of chemicals. Nucleic acids Res. 46 (W1), W257–W263. 10.1093/nar/gky318 29718510 PMC6031011

[B8] Bergdorf M. Kim E. T. Rendleman C. A. Shaw D. E. (2014). Desmond/GPU performance as of november 2014.

[B9] Bray F. F. Ferlay J. Soerjomataram I. Siegel R. L. Torre L. A. Jemal A. J. (2020). Erratum: global cancer statistics 2018: GLOBOCAN estimates of incidence and mortality worldwide for 36 cancers in 185 countries. Ca Cancer J. Clin. 70 (4), 313. 10.3322/caac.21609 30207593

[B10] Brown N. R. Korolchuk S. Martin M. P. Stanley W. A. Moukhametzianov R. Noble M. E. (2015). CDK1 structures reveal conserved and unique features of the essential cell cycle CDK. Nat. Commun. 6 (1), 6769. 10.1038/ncomms7769 25864384 PMC4413027

[B11] Casagrande F. Darbon J. M. (2001). Effects of structurally related flavonoids on cell cycle progression of human melanoma cells: regulation of cyclin-dependent kinases CDK2 and CDK111Abbreviations: CDK, cyclin-dependent kinase; CKI, CDK inhibitor; PI 3-kinase, phosphatidylinositol 3-kinase; PKC, protein kinase C; DTT, dithiothreitol; RIPA, radioimmunoprecipitation assay buffer. Biochem. Pharmacol. 61 (10), 1205–1215. 10.1016/s0006-2952(01)00583-4 11322924

[B12] Castrosanto M. A. Abrera A. T. Manalo M. N. Ghosh A. (2023). *In silico* evaluation of binding of phytochemicals from bayati (Anamirta cocculus Linn) to the glutathione-s-transferase of Asian Corn Borer (*Ostrinia furnacalis* Guenée). J. Biomol. Struct. Dyn. 41 (7), 2660–2666. 10.1080/07391102.2022.2036240 35138221

[B13] Cheng F. Li W. Zhou Y. Shen J. Wu Z. Liu G. (2022). admetSAR: a comprehensive source and free tool for assessment of chemical ADMET properties. 10.1021/ci300367a 23092397

[B14] Cheng T. Pan Y. Hao M. Wang Y. Bryant S. H. (2014). PubChem applications in drug discovery: a bibliometric analysis. Drug Discov. today 19 (11), 1751–1756. 10.1016/j.drudis.2014.08.008 25168772 PMC4252728

[B15] Chikwambi Z. Hidjo M. Chikondowa P. Afolabi L. Aketch V. Jayeoba G. (2023). BioRxiv.Multi-omics data integration approach identifies potential biomarkers for Prostate cancer

[B16] Choudhary M. I. Shaikh M. tul-Wahab A. ur-Rahman A. (2020). *In silico* identification of potential inhibitors of key SARS-CoV-2 3CL hydrolase (Mpro) via molecular docking, MMGBSA predictive binding energy calculations, and molecular dynamics simulation. Plos one 15 (7), e0235030. 10.1371/journal.pone.0235030 32706783 PMC7380638

[B17] Choudhury C. Murugan N. A. Priyakumar U. D. (2022). Structure-based drug repurposing: traditional and advanced AI/ML-aided methods. Drug Discov. Today 27, 1847–1861. 10.1016/j.drudis.2022.03.006 35301148 PMC8920090

[B18] Chow E. Rendleman C. A. Bowers K. J. Dror R. O. Hughes D. H. Gullingsrud J. (2008). Desmond performance on a cluster of multicore processors. China, DE Shaw Research Technical Report DESRES/TR--2008-01.

[B19] Cob-Calan N. N. Chi-Uluac L. A. Ortiz-Chi F. Cerqueda-García D. Navarrete-Vázquez G. Ruiz-Sánchez E. (2019). Molecular docking and dynamics simulation of protein β-tubulin and antifungal cyclic lipopeptides. Molecules 24 (18), 3387. 10.3390/molecules24183387 31540347 PMC6767525

[B20] Colomer R. Alba E. González-Martin A. Paz-Ares L. Martin M. Llombart A. (2010). Treatment of cancer with oral drugs: a position statement by the Spanish Society of Medical Oncology (SEOM). Ann. Oncol. 21 (2), 195–198. 10.1093/annonc/mdp595 20110291 PMC2813309

[B92] Coutsias E. A. Seok C. Dill K. A (2004). Using quaternions to calculate RMSD. J. Comput. Chem. 25 (15), 1849–1857. 10.1002/jcc.20110 15376254

[B21] Cragg G. M. Newman D. J. (2013). Natural products: a continuing source of novel drug leads. Biochimica Biophysica Acta (BBA)-General Subj. 1830 (6), 3670–3695. 10.1016/j.bbagen.2013.02.008 PMC3672862 23428572

[B22] Daina A. Michielin O. Zoete V. (2017). SwissADME: a free web tool to evaluate pharmacokinetics, drug-likeness and medicinal chemistry friendliness of small molecules. Sci. Rep. 7 (1), 42717. 10.1038/srep42717 28256516 PMC5335600

[B23] Dar R. A. Shahnawaz M. Ahanger M. A. ul Majid I. (2023). Exploring the diverse bioactive compounds from medicinal plants: a review. A Rev. 12, 189–195. 10.31254/phyto.2023.12307

[B24] Deep A. Marwaha R. K. Marwaha M. G. Nandal R. Sharma A. K. (2018). Flavopiridol as cyclin dependent kinase (CDK) inhibitor: a review. New J. Chem. 42 (23), 18500–18507. 10.1039/C8NJ04306J

[B25] Doak B. C. Over B. Giordanetto F. Kihlberg J. (2014). Oral druggable space beyond the rule of 5: insights from drugs and clinical candidates. Chem. Biol. 21, 1115–1142. 10.1016/j.chembiol.2014.08.013 25237858

[B26] Durrant J. D. McCammon J. A. (2011). Molecular dynamics simulations and drug discovery. BMC Biol. 9 (1), 71–79. 10.1186/1741-7007-9-71 22035460 PMC3203851

[B27] El Abed F. Baraket G. Nyamari M. N. Naitore C. Awe O. I. (2023). Differential expression analysis of miRNAs and mRNAs in epilepsy uncovers potential biomarkers. bioRxiv. 10.1101/2023.09.11.557132

[B28] Epik S. (2017a). Scrodinger release 2017. New York, NY: LigPrep.

[B29] Epik S. (2017b). Impact, schrodinger. New York, NY: LLC.

[B30] Ferlay J. Soerjomataram I. Dikshit R. Eser S. Mathers C. Rebelo M. (2015). Cancer incidence and mortality worldwide: sources, methods and major patterns in GLOBOCAN 2012. Int. J. Cancer 136 (5), E359–E386. 10.1002/ijc.29210 25220842

[B31] Friesner R. A. Murphy R. B. Repasky M. P. Frye L. L. Greenwood J. R. Halgren T. A. (2006). Extra precision glide: docking and scoring incorporating a model of hydrophobic enclosure for protein− ligand complexes. J. Med. Chem. 49 (21), 6177–6196. 10.1021/jm051256o 17034125

[B32] Gilson M. K. Given J. A. Bush B. L. McCammon J. A. (1997). The statistical-thermodynamic basis for computation of binding affinities: a critical review. Biophys. J. 72 (3), 1047–1069. 10.1016/S0006-3495(97)78756-3 9138555 PMC1184492

[B33] Gozalbes R. Pineda-Lucena A. (2011). Small molecule databases and chemical descriptors useful in chemoinformatics: an overview. Comb. Chem. high throughput Screen. 14 (6), 548–558. 10.2174/138620711795767857 21521149

[B34] Gralow J. Ozols R. F. Bajorin D. F. Cheson B. D. Sandler H. M. Winer E. P. (2008). Clinical cancer advances 2007: major research advances in cancer treatment, prevention, and screening—a report from the American Society of Clinical Oncology. J. Clin. Oncol. 26 (2), 313–325. 10.1200/JCO.2007.15.4088 18086794

[B35] Greenlee J. D. Liu K. Lopez-Cavestany M. King M. R. (2022). Piezo1 mechano-activation is augmented by resveratrol and differs between colorectal cancer cells of primary and metastatic origin. Molecules 27 (17), 5430. 10.3390/molecules27175430 36080197 PMC9458129

[B36] Guan L. Yang H. Cai Y. Sun L. Di P. Li W. (2019). ADMET-score–a comprehensive scoring function for evaluation of chemical drug-likeness. Medchemcomm 10 (1), 148–157. 10.1039/c8md00472b 30774861 PMC6350845

[B37] Han Y. Zhang J. Hu C. Q. Zhang X. Ma B. Zhang P. (2019). *In silico* ADME and toxicity prediction of ceftazidime and its impurities. Front. Pharmacol. 10, 434. 10.3389/fphar.2019.00434 31068821 PMC6491819

[B38] Harvey A. L. Edrada-Ebel R. Quinn R. J. (2015). The re-emergence of natural products for drug discovery in the genomics era. Nat. Rev. drug Discov. 14 (2), 111–129. 10.1038/nrd4510 25614221

[B39] He F. Wang X. Wu Q. Liu S. Cao Y. Guo X. (2023). Identification of potential ATP-competitive cyclin-dependent kinase 1 inhibitors: *de novo* drug generation, molecular docking, and molecular dynamics simulation. Comput. Biol. Med. 155, 106645. 10.1016/j.compbiomed.2023.106645 36774892

[B40] Holowatyj A. N. Maude A. S. Musa H. S. Adamu A. Ibrahim S. Abdullahi A. (2020). Patterns of early-onset colorectal cancer among Nigerians and African Americans. JCO Glob. Oncol. 6, 1647–1655. 10.1200/GO.20.00272 33141623 PMC7713583

[B41] Huang M. Lu J. J. Ding J. (2021). Natural products in cancer therapy: past, present and future. Nat. Prod. Bioprospect. 11, 5–13. 10.1007/s13659-020-00293-7 33389713 PMC7933288

[B42] Huang S. Y. Zou X. (2010). Advances and challenges in protein-ligand docking. Int. J. Mol. Sci. 11 (8), 3016–3034. 10.3390/ijms11083016 21152288 PMC2996748

[B43] Ikwu F. A. Isyaku Y. Obadawo B. S. Lawal H. A. Ajibowu S. A. (2020). *In silico* design and molecular docking study of CDK2 inhibitors with potent cytotoxic activity against HCT116 colorectal cancer cell line. J. Genet. Eng. Biotechnol. 18 (1), 51–52. 10.1186/s43141-020-00066-2 32930901 PMC7492310

[B44] Jiang Z. You L. Dou W. Sun T. Xu P. (2019). Effects of an electric field on the conformational transition of the protein: a molecular dynamics simulation study. Polymers 11 (2), 282. 10.3390/polym11020282 30960266 PMC6419079

[B45] Kalani K. Agarwal J. Alam S. Khan F. Pal A. Srivastava S. K. (2013). *In silico* and *in vivo* anti-malarial studies of 18β glycyrrhetinic acid from Glycyrrhiza glabra. PloS one 8 (9), e74761. 10.1371/journal.pone.0074761 24086367 PMC3782471

[B46] Kalous J. Jansová D. Šušor A. (2020). Role of cyclin-dependent kinase 1 in translational regulation in the M-phase. Cells 9 (7), 1568. 10.3390/cells9071568 32605021 PMC7408968

[B47] Kollman P. A. Massova I. Reyes C. Kuhn B. Huo S. Chong L. (2000). Calculating structures and free energies of complex molecules: combining molecular mechanics and continuum models. Accounts Chem. Res. 33 (12), 889–897. 10.1021/ar000033j 11123888

[B48] Li J. Wang Y. Wang X. Yang Q. (2020). CDK1 and CDC20 overexpression in patients with colorectal cancer are associated with poor prognosis: evidence from integrated bioinformatics analysis. World J. Surg. Oncol. 18 (1), 50–51. 10.1186/s12957-020-01817-8 32127012 PMC7055103

[B49] Li Q. Cheng T. Wang Y. Bryant S. H. (2010). PubChem as a public resource for drug discovery. Drug Discov. today 15 (23-24), 1052–1057. 10.1016/j.drudis.2010.10.003 20970519 PMC3010383

[B50] Liao H. Ji F. Ying S. (2017). CDK1: beyond cell cycle regulation. Aging (Albany NY) 9 (12), 2465–2466. 10.18632/aging.101348 29242409 PMC5764383

[B51] Lipinski C. A. Lombardo F. Dominy B. W. Feeney P. J. (1997). Experimental and computational approaches to estimate solubility and permeability in drug discovery and development settings. Adv. drug Deliv. Rev. 23 (1-3), 3–25. 10.1016/S0169-409X(96)00423-1 11259830

[B52] Lobanov M. Y. Bogatyreva N. S. Galzitskaya O. V. (2008). Radius of gyration as an indicator of protein structure compactness. Mol. Biol. 42, 623–628. 10.1134/s0026893308040195 18856071

[B53] Manallack D. T. Prankerd R. J. Yuriev E. Oprea T. I. Chalmers D. K. (2013). The significance of acid/base properties in drug discovery. Chem. Soc. Rev. 42 (2), 485–496. 10.1039/c2cs35348b 23099561 PMC3641858

[B54] Massova I. Kollman P. A. (2000). Combined molecular mechanical and continuum solvent approach (MM-PBSA/GBSA) to predict ligand binding. Perspect. drug Discov. Des. 18, 113–135. 10.1023/a:1008763014207

[B55] Matthews H. K. Bertoli C. de Bruin R. A. (2022). Cell cycle control in cancer. Nat. Rev. Mol. Cell. Biol. 23 (1), 74–88. 10.1038/s41580-021-00404-3 34508254

[B56] McGrath C. F. Pattabiraman N. Kellogg G. E. Lemcke T. Kunick C. Sausville E. A. (2005). Homology model of the CDK1/cyclin B complex. J. Biomol. Struct. Dyn. 22 (5), 493–502. 10.1080/07391102.2005.10531227 15702922

[B57] Navarro-Retamal C. Caballero J. (2016). Flavonoids as CDK1 inhibitors: insights in their binding orientations and structure-activity relationship. PLoS One 11 (8), e0161111. 10.1371/journal.pone.0161111 27517610 PMC4982677

[B58] Newman D. J. Cragg G. M. (2016). Natural products as sources of new drugs from 1981 to 2014. J. Nat. Prod. 79 (3), 629–661. 10.1021/acs.jnatprod.5b01055 26852623

[B59] Nicolini F. Burmistrova O. Marrero M. T. Torres F. Hernández C. Quintana J. (2014). Induction of G2/M phase arrest and apoptosis by the flavonoid tamarixetin on human leukemia cells. Mol. Carcinog. 53 (12), 939–950. 10.1002/mc.22055 23765509

[B60] Nyamari M. N. Omar K. M. Fayehun A. F. Dachi O. Bwana B. K. Awe O. I. (2023). Expression level analysis of ACE2 receptor gene in african-American and non-african-American COVID-19 patients. bioRxiv. 10.1101/2023.09.11.557129

[B61] Nzungize L. Kengne-Ouafo J. A. Wesonga M. R. Umuhoza D. Murithi K. Kimani P. (2022). Transcriptional profiles analysis of COVID-19 and malaria patients reveals potential biomarkers in children. bioRxiv, 2022.06.30.498338. 10.1101/2022.06.30.498338

[B62] Rathod S. Shinde K. Porlekar J. Choudhari P. Dhavale R. Mahuli D. (2022). Computational exploration of anti-cancer potential of flavonoids against cyclin-dependent kinase 8: an *in silico* molecular docking and dynamic approach. ACS omega 8, 391–409. 10.1021/acsomega.2c04837 36643495 PMC9835631

[B63] Ravishankar D. Rajora A. K. Greco F. Osborn H. M. (2013). Flavonoids as prospective compounds for anti-cancer therapy. Int. J. Biochem. Cell. Biol. 45 (12), 2821–2831. 10.1016/j.biocel.2013.10.004 24128857

[B64] Rehman Y. Rosenberg J. E. (2012). Abiraterone acetate: oral androgen biosynthesis inhibitor for treatment of castration-resistant prostate cancer. Drug Des. Dev. Ther. 6, 13–18. 10.2147/DDDT.S15850 PMC3267518 22291466

[B65] Release S. 1: Desmond molecular dynamics system. DE Shaw Research, New York, NY, Maestro-desmond interoperability tools, version. 2014;3.

[B66] Roberts R. A. Kavanagh S. L. Mellor H. R. Pollard C. E. Robinson S. Platz S. J. (2014). Reducing attrition in drug development: smart loading preclinical safety assessment. Drug Discov. today 19 (3), 341–347. 10.1016/j.drudis.2013.11.014 24269835

[B67] Saikat A. S. (2021). An *in silico* approach for potential natural compounds as inhibitors of protein CDK1/cks2. Chem. Proc. 8 (1), 5. 10.3390/ecsoc-25-11721

[B68] Salmaso V. Moro S. (2018). Bridging molecular docking to molecular dynamics in exploring ligand-protein recognition process: an overview. Front. Pharmacol. 9, 923. 10.3389/fphar.2018.00923 30186166 PMC6113859

[B69] Sarkar I. Sen A. (2022). *In silico* screening predicts common cold drug Dextromethorphan along with Prednisolone and Dexamethasone can be effective against novel Coronavirus disease (COVID-19). J. Biomol. Struct. Dyn. 40 (8), 3706–3710. 10.1080/07391102.2020.1850528 33225870 PMC7754926

[B70] Sasidharan S. Chen Y. Saravanan D. Sundram K. M. Latha L. Y. (2011). Extraction, isolation and characterization of bioactive compounds from plants’ extracts. Afr. J. traditional, complementary Altern. Med. 8 (1), 1–10. 10.4314/ajtcam.v8i1.60483 PMC3218439 22238476

[B71] Sastry M. G. Adzhigirey M. Day T. Annabhimoju R. Sherman W. (2013). Protein and ligand preparation: parameters, protocols, and influence on virtual screening enrichments. J. computer-aided Mol. Des. 27, 221–234. 10.1007/s10822-013-9644-8 23579614

[B72] Schmidt M. Rohe A. Platzer C. Najjar A. Erdmann F. Sippl W. (2017). Regulation of G2/M transition by inhibition of WEE1 and PKMYT1 kinases. Molecules 22 (12), 2045. 10.3390/molecules22122045 29168755 PMC6149964

[B73] Segall M. D. Barber C. (2014). Addressing toxicity risk when designing and selecting compounds in early drug discovery. Drug Discov. today 19 (5), 688–693. 10.1016/j.drudis.2014.01.006 24451294

[B74] Sherman W. Beard H. S. Farid R. (2006). Use of an induced fit receptor structure in virtual screening. Chem. Biol. drug Des. 67 (1), 83–84. 10.1111/j.1747-0285.2005.00327.x 16492153

[B75] Simon L. Imane A. Srinivasan K. K. Pathak L. Daoud I. (2017). *In silico* drug-designing studies on flavanoids as anticolon cancer agents: pharmacophore mapping, molecular docking, and Monte Carlo method-based QSAR modeling. Interdiscip. Sci. Comput. Life Sci. 9, 445–458. 10.1007/s12539-016-0169-4 27059855

[B76] Sofi S. Mehraj U. Qayoom H. Aisha S. Almilaibary A. Alkhanani M. (2022a). Targeting cyclin-dependent kinase 1 (CDK1) in cancer: molecular docking and dynamic simulations of potential CDK1 inhibitors. Med. Oncol. 39 (9), 133. 10.1007/s12032-022-01748-2 35723742 PMC9207877

[B77] Sofi S. Mehraj U. Qayoom H. Aisha S. Asdaq S. M. Almilaibary A. (2022b). Cyclin-dependent kinases in breast cancer: expression pattern and therapeutic implications. Med. Oncol. 39 (6), 106. 10.1007/s12032-022-01731-x 35486263

[B78] Tripathi N. Goel B. Bhardwaj N. Sahu B. Kumar H. Jain S. K. (2022). Virtual screening and molecular simulation study of natural products database for lead identification of novel coronavirus main protease inhibitors. J. Biomol. Struct. Dyn. 40 (8), 3655–3667. 10.1080/07391102.2020.1848630 33213294

[B79] Tripathi S. K. Muttineni R. Singh S. K. (2013). Extra precision docking, free energy calculation and molecular dynamics simulation studies of CDK2 inhibitors. J. Theor. Biol. 334, 87–100. 10.1016/j.jtbi.2013.05.014 23727278

[B80] Tripathi S. K. Singh S. K. Singh P. Chellaperumal P. Reddy K. K. Selvaraj C. (2012). Exploring the selectivity of a ligand complex with CDK2/CDK1: a molecular dynamics simulation approach. J. Mol. Recognit. 25 (10), 504–512. 10.1002/jmr.2216 22996593

[B81] Tsui V. Case D. A. (2000). Theory and applications of the generalized Born solvation model in macromolecular simulations. Biopolymers Orig. Res. Biomol. 56 (4), 275–291. 10.1002/1097-0282(2000)56:4<275::aid-bip10024>3.0.co;2-e 11754341

[B82] Veber D. F. Johnson S. R. Cheng H. Y. Smith B. R. Ward K. W. Kopple K. D. (2002). Molecular properties that influence the oral bioavailability of drug candidates. J. Med. Chem. 45 (12), 2615–2623. 10.1021/jm020017n 12036371

[B83] Wan H. (2013). What ADME tests should be conducted for preclinical studies? ADMET DMPK 1 (3), 19–28. 10.5599/admet.1.3.9

[B84] Weitz J. Koch M. Debus J. Höhler T. Galle P. R. Büchler M. W. (2005). Colorectal cancer. Lancet 365 (9454), 153–165. 10.1016/s0140-6736(05)17706-x 15639298

[B85] Xu M. Lill M. A. (2013). Induced fit docking, and the use of QM/MM methods in docking. Drug Discov. Today Technol. 10 (3), e411–e418. 10.1016/j.ddtec.2013.02.003 24050138 PMC3719877

[B86] Yang H. Lou C. Sun L. Li J. Cai Y. Wang Z. (2019). admetSAR 2.0: web-service for prediction and optimization of chemical ADMET properties. Bioinformatics 35 (6), 1067–1069. 10.1093/bioinformatics/bty707 30165565

[B87] Yilmaz Goler A. M. Jannuzzi A. T. Bayrak N. Yıldız M. Yıldırım H. Otsuka M. (2022). *In vitro* and *in silico* study to assess toxic mechanisms of hybrid molecules of quinone-benzocaine as plastoquinone analogues in breast cancer cells. ACS omega 7 (34), 30250–30264. 10.1021/acsomega.2c03428 36061710 PMC9434764

[B93] Yuan X. H. Wang Y. C. Jin W. J. Zhao B. B. Chen C. F. Yang J. (2012). Structure-based high-throughput epitope analysis of hexon proteins in B and C species human adenoviruses (HAdVs). PLoS One 7 (3), e32938. 10.1371/journal.pone.0032938 22427913 PMC3302796

[B88] Zhang L. Zhu H. Wang Q. Fang H. Xu W. Li M. (2011). Homology modeling, molecular dynamic simulation and docking studies of cyclin dependent kinase 1. J. Mol. Model. 17, 219–226. 10.1007/s00894-010-0710-z 20419498

[B89] Zhu Y. Bian Y. Zhang Q. Hu J. Li L. Yang M. (2019). Construction and analysis of dysregulated lncRNA‐associated ceRNA network in colorectal cancer. J. Cell. Biochem. 120 (6), 9250–9263. 10.1002/jcb.28201 30525245

[B90] Zhu Y. Bian Y. Zhang Q. Hu J. Li L. Yang M. (2020b). LINC00365 promotes colorectal cancer cell progression through the Wnt/β‐catenin signaling pathway. J. Cell. Biochem. 121 (2), 1260–1272. 10.1002/jcb.29359 31544991

[B91] Zhu Y. Li K. Zhang J. Wang L. Sheng L. Yan L. (2020a). Inhibition of CDK1 reverses the resistance of 5-Fu in colorectal cancer. Cancer Manag. Res. 12, 11271–11283. 10.2147/cmar.s255895 33177877 PMC7649235

